# *Sb*-Phenyl-*N*-methyl-5,6,7,12-tetrahydrodibenz[*c,f*][1,5]azastibocine Induces Perlecan Core Protein Synthesis in Cultured Vascular Endothelial Cells

**DOI:** 10.3390/ijms24043656

**Published:** 2023-02-11

**Authors:** Takato Hara, Tomoko Konishi, Shuji Yasuike, Yasuyuki Fujiwara, Chika Yamamoto, Toshiyuki Kaji

**Affiliations:** 1 Faculty of Pharmaceutical Sciences, Toho University, 2-2-1 Miyama, Funabashi, Chiba 274-8510, Japan; 2 Faculty of Pharmaceutical Sciences, Hokuriku University, Ho-3 Kanagawa-machi, Kanazawa, Ishikawa 920-1181, Japan; 3 School of Pharmaceutical Sciences, Aichi Gakuin University, 1-100 Kusumoto-cho, Chikusa-ku, Nagoya, Aichi 464-8650, Japan; 4 School of Pharmacy, Tokyo University of Pharmacy and Life Sciences, 1432 Horinouchi, Hachioji, Tokyo 192-0392, Japan; 5 Faculty of Pharmaceutical Sciences, Tokyo University of Science, 2641 Yamazaki, Noda, Chba 278-8510, Japan

**Keywords:** organoantimony, vascular endothelial cells, proteoglycan, core protein, perlecan

## Abstract

Vascular endothelial cells synthesize and secrete perlecan, a large heparan sulfate proteoglycan that increases the anticoagulant activity of vascular endothelium by inducing antithrombin III and intensifying fibroblast growth factor (FGF)-2 activity to promote migration and proliferation in the repair process of damaged endothelium during the progression of atherosclerosis. However, the exact regulatory mechanisms of endothelial perlecan expression remain unclear. Since organic–inorganic hybrid molecules are being developed rapidly as tools to analyze biological systems, we searched for a molecular probe to analyze these mechanisms using a library of organoantimony compounds and found that the *Sb*-phenyl-*N*-methyl-5,6,7,12-tetrahydrodibenz[*c,f*][1,5]azastibocine (PMTAS) molecule promotes the expression of perlecan core protein gene without exhibiting cytotoxicity in vascular endothelial cells. In the present study, we characterized proteoglycans synthesized by cultured bovine aortic endothelial cells using biochemical techniques. The results indicated that PMTAS selectively induced perlecan core protein synthesis, without affecting the formation of its heparan sulfate chain, in vascular endothelial cells. The results also implied that this process is independent of the endothelial cell density, whereas in vascular smooth muscle cells, it occurred only at high cell density. Thus, PMTAS would be a useful tool for further studies on the mechanisms underlying perlecan core protein synthesis in vascular cells, which is critical in the progression of vascular lesions, such as those during atherosclerosis.

## 1. Introduction

Organic–inorganic hybrid molecules are those composed of metal(s) and organic components. Hybrid molecules are being developed rapidly as tools to analyze biological systems [1]. Our research group has reported several functional hybrid molecules, including 2,9-dimethyl-1,10-phenanthroline zinc that enhances the growth of vascular endothelial cells [2] and tris(pentafluorophenyl)stibine that was used to reveal the induction of metallothionein subisoforms via different intracellular signal pathways [3], and copper diethyldithiocarbamate that induces the expression of endothelial syndecan-4 via the p38 mitogen-activated protein kinase (MAPK) pathway [4], as well as that of reactive sulfur species producing the enzyme cystathionine γ-lyase via multiple signaling pathways [5].

The reactivity of hybrid molecules is unique and different from that of metallic compound(s) or organic structure(s). We found that the cytotoxicity of organic–inorganic hybrid molecules was difficult to predict. Copper is an essential trace element with low cytotoxicity; however, it exhibited strong cytotoxicity when being bound to the nontoxic ligand 2,9-dimethyl-1,10-phenanthroline in vascular endothelial cells [4]. An organoantimony compound *Sb*-phenyl-*N*-methyl-5,6,7,12-tetrahydrodibenz[*c,f*][1,5]azastibocine (PMTAS) is nontoxic; however, it exhibited cytotoxicity in vascular endothelial cells following the substitution of its antimony atom with the otherwise nontoxic inorganic bismuth [6,7]. The cytotoxicity of pentavalent organoantimony compounds with three phenyl groups has been shown to be not correlated with their hydrophobicity or intracellular accumulation [8]. In bis(1,4-dihydro-2-methyl-1-phenyl-4-thioxo-3-pyridiolato)zinc(II), only the binding site of the metal atom is responsible for accumulation of zinc complexes, but the entire structure is responsible for their cytotoxicity in vascular endothelial cells [9]. Conclusively, it is suggested that organic–inorganic hybrid molecules exhibit biological activities as well as cytotoxic properties that are different from those of their component inorganic metals and organic structures.

Proteoglycans are macromolecules that consist of a core protein to which glycosaminoglycan(s) are covalently bound [10]. Proteoglycans in vascular endothelial cells and their extracellular matrix modulate various vascular functions, such as the blood coagulation-fibrinolytic system, permeability, and lipid metabolism [11,12,13,14]. Atherosclerosis is a chronic inflammatory disease caused by functional damage of vascular endothelial cells [15]. Endothelial proteoglycans, owing to their involvement in lipid metabolism and the blood coagulation system, are one of the key molecules contributing to the progression of atherosclerosis [16]. Vascular endothelial cells synthesize two types of proteoglycans: heparan sulfate and dermatan sulfate proteoglycans. The large proteoglycan perlecan is a type of heparan sulfate proteoglycan secreted from the cells and is a major component of the basement membrane [17]. The transmembrane syndecan family members, including syndecan-1 and -4, are also other proteoglycans in this group [18]. An examples of a dermatan sulfate proteoglycan includes biglycan, a secreted small leucine-rich dermatan sulfate proteoglycan [19]. Among the proteoglycans, perlecan possesses heparan sulfate chains and exhibits a higher antithrombin III activating activity [20]. Vascular endothelial cells release fibroblast growth factor-2 (FGF-2) when there is cell damage [21]. The FGF receptors on the cells near the damage site are activated by formation of a ternary complex of FGF-2, FGF receptors, and perlecan, which promotes the migration and proliferation of the cells during repair [22].

As stated above, perlecan plays important roles for the anticolagulant property and promotion of the repair process by FGF-2 in vascular endothelium. However, the regulatory mechanisms of the expression of proteoglycans, specifically perlecan, in vascular endothelial cells have not been fully elucidated. In order to analyze the detailed mechanisms underlying endothelial perlecan synthesis, we believe that molecular probes that induce endothelial perlecan synthesis are required to resolve the obscurity. In the previous study, we reported a small molecule that can modulate endothelial proteoglycan species such as diethyldithiocarbamate copper(II) and 1,10-phenanthroline, both of which induce endothelial syndecan-4 expression via the p38 MAPK and hypoxia-inducible factor (HIF)-1α pathways, respectively [23,24]. Additionally, we had recently reported a zinc complex dichloro(2,9-dimethyl-1,10-phenanthroline)zinc(II) that upregulates perlecan in a cell density dependent manner; however, this activity was due to its organic structure [25]. Therefore, we considered that an organoantimony compound PMTAS may affect endothelial perlecan synthesis owing to its nontoxicity. Hence, we also aimed to clarify the effect of PMTAS on proteoglycan synthesis in vascular endothelial cells.

## 2. Results

### 2.1. PMTAS Enhances [^35^S]sulfate Incorporation into Proteoglycans in Vascular Endothelial Cells

**Figure 1:**
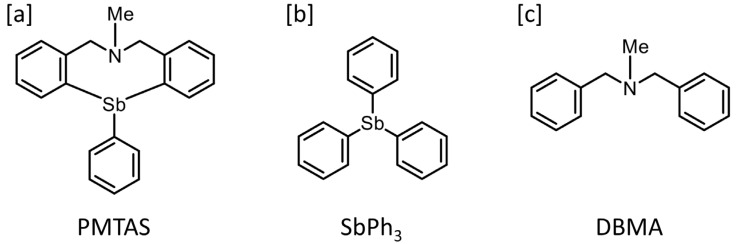
The structures of PMTAS (**a**); SbPh_3_ (**b**); and DBMA (**c**).

By a DNA microarray analysis, we found that PMTAS is an organoantimony compound that elevates the expression of perlecan mRNA without exhibiting cytotoxicity in vascular endothelial cells from a library of 24 organoantimony compounds (the structures and molecular forms/names are shown in Figure S1 and Table S1, respectively). The structures of PMTAS, triphenylstibine (SbPh_3_) and *N,N*-dibenzylmethylamine (DBMA) are shown in Figure 1. The structures of SbPh_3_ and DBMA can be derived from that of PMTAS by eliminating the bridge containing the nitrogen and the bismuth atoms, respectively.

**Figure 2:**
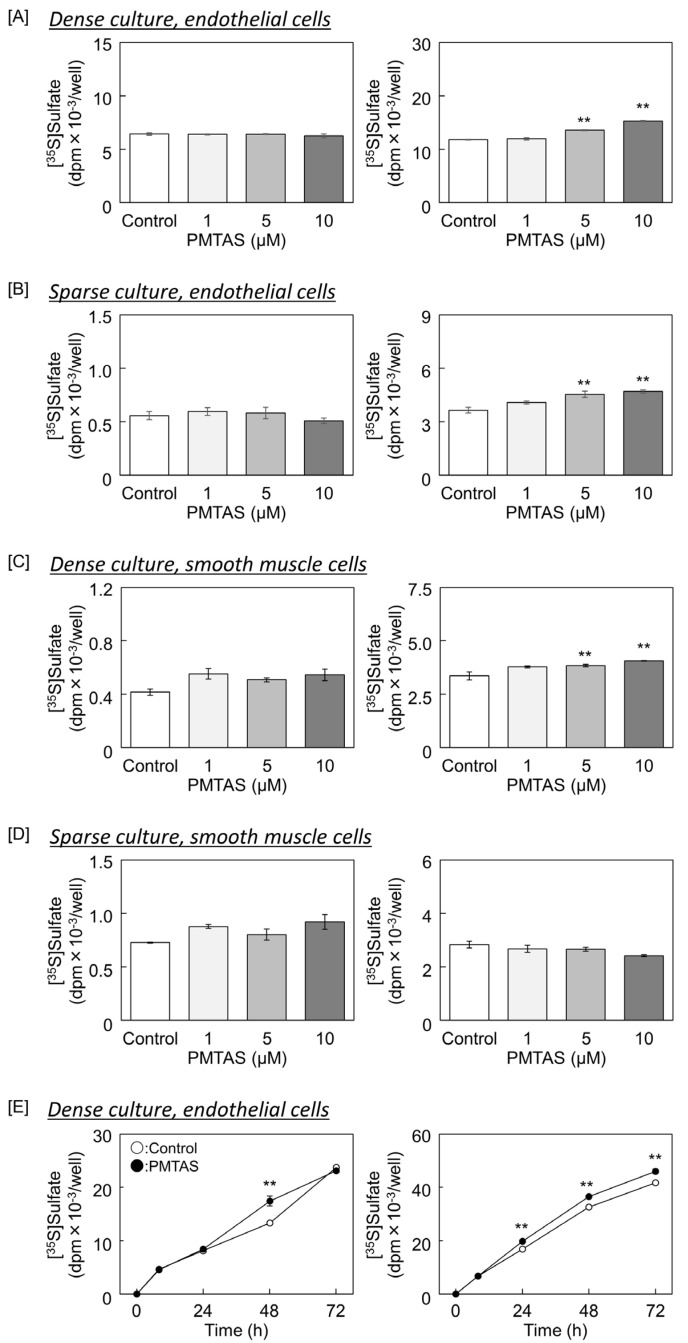
[^35^S]Sulfate incorporation into the proteoglycans that accumulated in the cell layer (**left panels**) and conditioned medium (**right panels**). (**A**–**D**) Dense and sparse cultures of vascular endothelial and smooth muscle cells treated with PMTAS (1, 5, and 10 µM) for 24 h; (**E**) or with PMTAS 10 µM for 8, 24, 48, and 72 h. (**A**,**E**) Dense cultures of vascular endothelial cells, (**B**) sparse cultures of vascular endothelial cells, (**C**) dense cultures of vascular smooth muscle cells, (**D**) sparse culture of vascular smooth muscle cells. Values are means ± S.E. of three samples. ** *p* < 0.01 vs. the corresponding control.

First, we examined whether PMTAS affects the accumulation of proteoglycans synthesized by vascular endothelial cells in the cell layer and conditioned medium. In our system, perlecan and biglycan are detected in both the cell layer and conditioned medium, while syndecans accumulates only in the cell layer [26,27]. The accumulation of [^35^S]sulfate-labeled proteoglycans in the conditioned medium dose-dependently increased in a significant manner on treatment with PMTAS in both dense and sparse cultures of vascular endothelial cells (Figure 2A,B, respectively). However, in the case of vascular smooth muscle cells, PMTAS increased the accumulation of radiolabeled proteoglycans secreted into the conditioned medium only in the dense culture (Figure 2C,D). Proteoglycan accumulation in the cell layer was unaffected by treatment with PMTAS in both vascular endothelial and smooth muscle cells with high or low cell densities. Additionally, the amount of radiolabeled proteoglycans secreted by vascular endothelial cells increased between 24 and 72 h following PMTAS treatment (Figure 2E).

**Figure 3:**
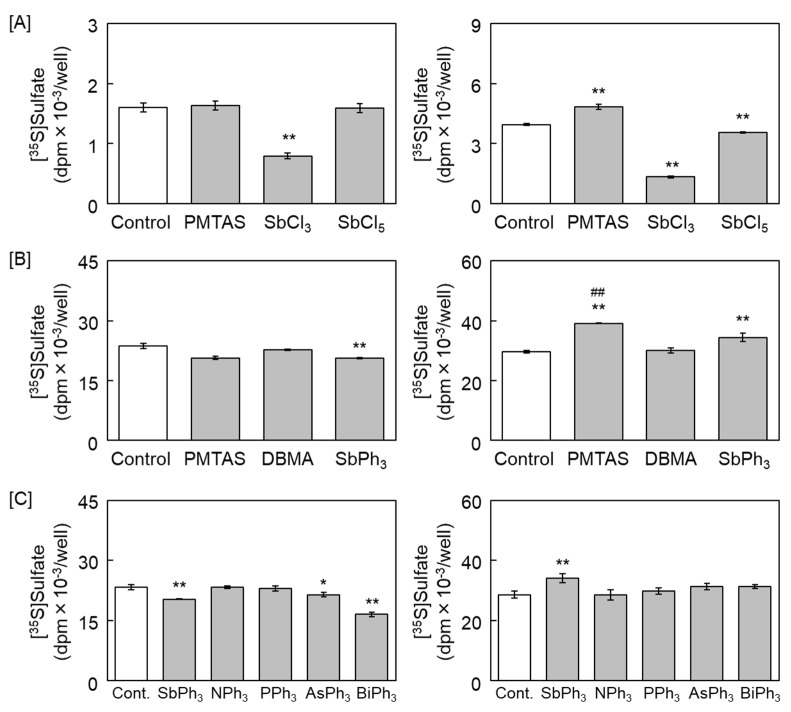
Comparison of the [^35^S]sulfated proteoglycans that accumulated in the cell layer (**left panels**) and conditioned medium (**right panels**) among the structural constitution of PMTAS: (**A**) Dense culture of vascular endothelial cells treated with PMTAS, SbCl_3_, and SbCl_5_ (10 µM each) for 24 h, values are means ± S.E. of three samples, ** *p* < 0.01 vs. control; (**B**) dense culture of vascular endothelial cells treated with PMTAS, DBMA, and SbPh_3_ (10 µM each) for 48 h, values are means ± S.E. of three samples, ** *p* < 0.01 vs. control and ^##^ *p* < 0.01 vs. SbPh_3_; (**C**) dense culture of vascular endothelial cells treated with SbPh_3_, NPh_3_, PPh_3_, AsPh, and BiPh_3_ (10 µM each) for 48 h, values are means ± S.E. of three samples, ** *p* < 0.01 and * *p* < 0.05 vs. control.

To clarify the roles of the partial structure of PMTAS, a triphenylantimony structure, and the bridge containing the nitrogen atom, in the enhancement of the accumulation of radiolabeled proteoglycans in the conditioned medium, vascular endothelial cells were treated with PMTAS, SbCl_3_, SbCl_5_, DBMA, or SbPh_3_. SbCl_3_ and SbCl_5_ both decreased the incorporation of [^35^S]sulfate into proteoglycans accumulated in both the cell layer and conditioned medium (Figure 3A). This decrease is attributed to the cytotoxicity of inorganic antimony [28]. SbPh_3_ enhanced the accumulation of [^35^S]sulfate-labeled proteoglycans in the conditioned medium slightly but remarkably, and such enhancement was not detected following treatment with DBMA. However, the accumulation level of [^35^S]sulfate-labeled proteoglycans by SbPh_3_ treatment was significantly lower than that of PMTAS (Figure 3B). Additionally, as shown in Figure 3C, among the homologous elements of SbPh_3_, including triphenylamine (NPh_3_), triphenylphosphine (PPh_3_), triphenylarsine (AsPh_3_), and triphenylbismuth (BiPh_3_), only SbPh_3_ significantly increased the accumulation of [^35^S]sulfate-labeled proteoglycans in the conditioned medium.

**Figure 4:**
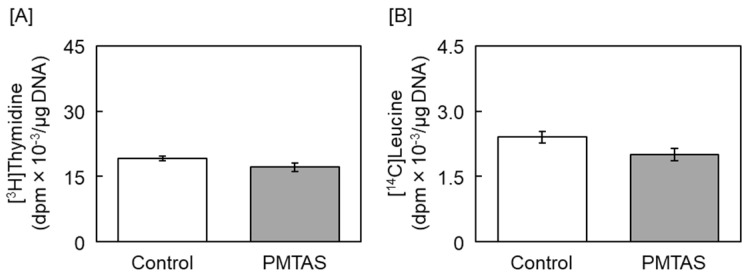
Effects of PMTAS on the incorporation of [^3^H]thymidine and [^14^C]leucine into the acid-insoluble fraction of vascular endothelial cells. The cells were treated with PMTAS (10 µM) for 24 h and labeled with (**A**) [^3^H]thymidine and (**B**) [^14^C]leucine during the last 6 h of treatment. Values are means ± S.E. of four samples.

The incorporation of both [^3^H]thymidine and [^14^C]leucine was unaffected by PMTAS (Figure 4), indicating that PMTAS does not affect either DNA or protein synthesis. This also indicates that enhancement of endothelial proteoglycan synthesis cannot be attributed to an increase in whole DNA and protein synthesis.

### 2.2. PMTAS Increases the Accumulation of Perlecan in the Conditioned Medium of Vascular Endothelial Cells

**Figure 5:**
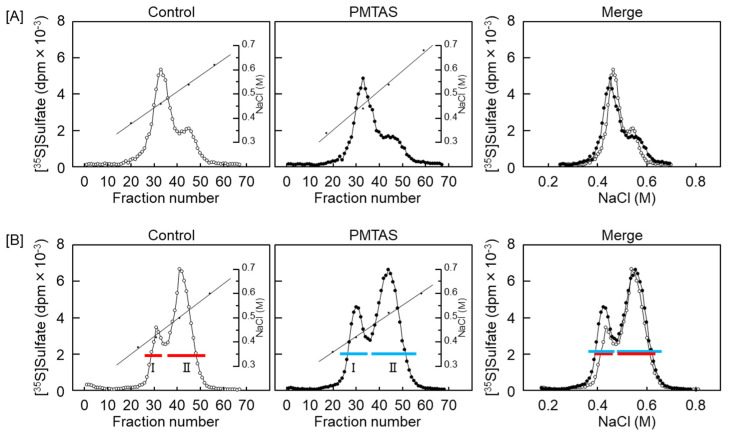
Characterization of proteoglycans based on negative charge density. Proteoglycan extraction from the (**A**) cell layer and (**B**) conditioned medium from dense culture of vascular endothelial cells treated with PMTAS (10 µM) for 48 h and separated via DEAE-Sephacel anion-exchange chromatography. The fractions within the range of red and blue horizontal bars are pooled as Peak I and Peak II.

**Figure 6:**
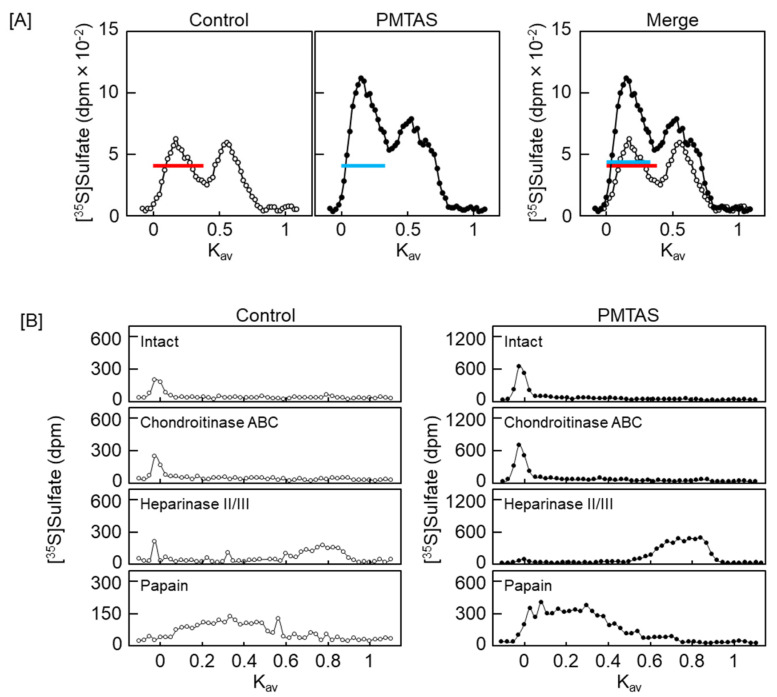
Characterization of proteoglycans based on hydrodynamic size: (**A**) High charged molecules pooled after DEAE-Sephacel anion-exchange chromatography were separated by Sepharose CL-4B molecular sieve chromatography. The fractions within the range of red and blue horizontal bars are pooled as high molecular weight subclass; (**B**) proteoglycans included in the high molecular weight subclass were digested with chondroitinase ABC, heparinase II/III, or papain, followed by separation via Sepharose CL-6B molecular sieve chromatography.

**Figure 7:**
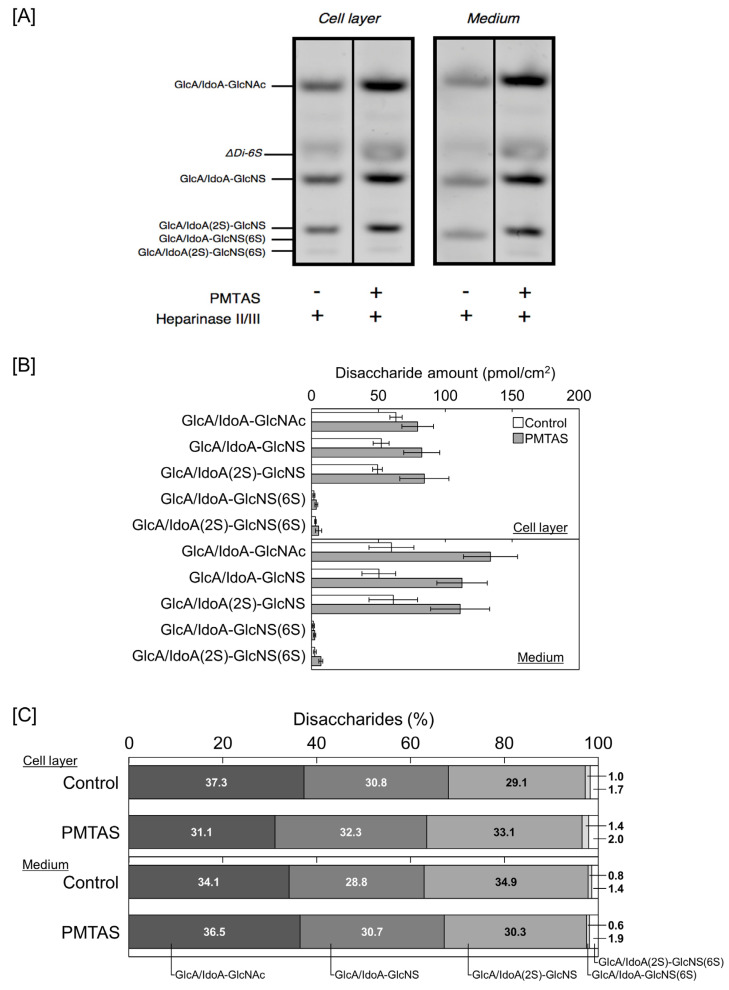
Fluorescence-assisted carbohydrate electrophoresis analysis of heparan sulfate accumulated in the cell layer and conditioned medium of dense cultures of vascular endothelial cells treated with PMTAS (10 µM) for 48 h: (**A**) A representative gel image; (**B**) disaccharide composition of heparan sulfate chains, values are means ± S.E. of four independent samples; (**C**) the percentage of the disaccharide units in heparan sulfate chains.

**Figure 8:**
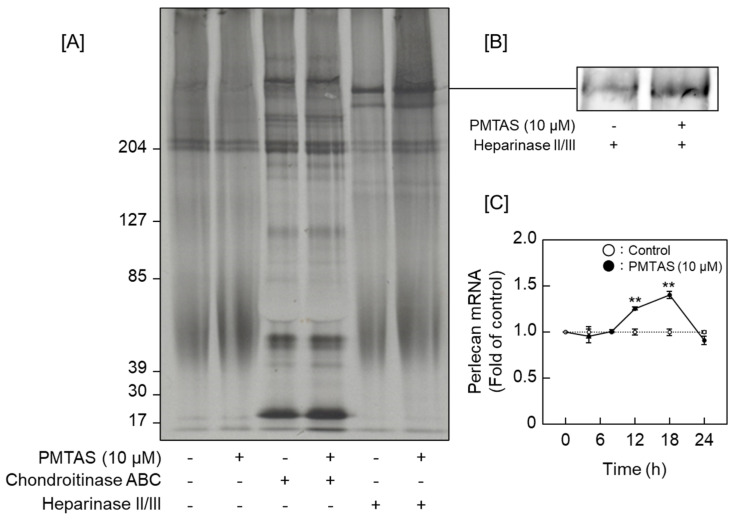
Expression levels of perlecan core protein that accumulated in the conditioned medium and perlecan mRNA in vascular endothelial cells after treatment with PMTAS: [**A**] [^35^S]Amino acids-labeled proteoglycan core proteins analysis via autoradiography, vascular endothelial cells were treated with PMTAS (10 µM) for 48 h; (**B**) perlecan core protein expression detection via Western blotting, vascular endothelial cells were treated with PMTAS (10 µM) for 48 h; (**C**) perlecan core protein mRNA expression, values are represented as means ± S.E. of four samples, ** *p* < 0.01 vs. corresponding control.

We subsequently investigated the characteristics of proteoglycans that were increased in the conditioned medium by PMTAS treatment. The [^35^S]sulfate-labeled molecules synthesized by vascular endothelial cells were separated based on their negative charge density via diethylaminoethyl (DEAE)-Sephacel ion-exchange chromatography. Radioactivity in each fraction of the cell layer did not vary significantly following the PMTAS treatment (Figure 5A). As shown in Figure 5B, there were two populations eluted at approximately 0.45 and 0.55 M NaCl in the conditioned medium (peak I and II, respectively). The radioactivity of peak II was not increased by PMTAS treatment. However, PMTAS increased the radioactivity of peak I. The molecules included in this peak were further separated via molecular sieve Sepharose CL-4B chromatography, which revealed the presence of two populations within peak I. Specifically, PMTAS increased [^35^S]sulfate incorporation into the high molecular weight subclass (Figure 6A). The molecule in this subclass was sensitive to digestion with both heparinase II/III and papain but not with chondroitinase ABC (Figure 6B). In other words, PMTAS enhanced the accumulation of a type of large heparan sulfate proteoglycans that were secreted into the conditioned medium from vascular endothelial cells. The characteristics of these secreted proteoglycans were consistent with those of perlecan. However, purification by DEAE-Sephacel and Sepharose CL-4B/CL-6B of [^35^S]sulfate-labeled proteoglycans revealed that PMTAS increased the synthesis of perlecan, a type of proteoglycan with a high molecular weight and heparin sulfate chains, not only selectively but also markedly in vascular endothelial cells, PMTAS markedly increased the [^35^S]sulfate incorporation. Additionally, we analyzed the effects of PMTAS on the disaccharide composition of heparan sulfate chains synthesized by vascular endothelial cells. As shown in Figure 7A,B, the disaccharides that constituted the heparan sulfate chains were mainly glucronic acid/iduronic acid-*N*-acetylglucosamine (GlcA/IdoA-GlcNAc), GlcA/IdoA-*N*-sulfated glucosamine (GlcA/IdoA-GlcNS), or 2-*O*-sulfated GlcA/IdoA-GlcNS (GlcA/IdoA(2S)-GlcNS); and importantly, PMTAS increased the quantity of these disaccharides. However, the content ratio of these disaccharide units in heparan sulfate chains was not significantly affected by PMTAS (Figure 7C). Unsaturated GlcA/IdoA-6-*O*-sulfated GlcNS (GlcA/IdoA-GlcNS(6S)) and GlcA/IdoA(2S)-GlcNS(6S) were detected only slightly. Furthermore, we examined the proteoglycan upregulation by PMTAS treatment in vascular endothelial cells. To support the results on characterization of proteoglycans shown in Figure 5 and Figure 6, PMTAS selectively induced the expression of perlecan core protein, which was induced from the mRNA level of vascular endothelial cells (Figure 8).

## 3. Discussion

Bio-organometallics is a biological research strategy using organic–inorganic hybrid molecules as a molecular probe to analyze biological systems [1]. As the mechanisms underlying the synthesis of perlecan in vascular endothelial cells have not been fully understood, finding molecular probes to elucidate these mechanisms are of great research interest. Endothelial perlecan expression is known to be induced by several cytokines/growth factors such as vascular endothelial growth factor (VEGF) [29], transforming growth factor (TGF)-β_1_ [26], and interleukin (IL)-1α [30,31]. VEGF-A165 induces the synthesis of perlecan with shorter heparan sulfate side chains in cultured human brain microvascular endothelial cells; however, the disaccharide composition of the heparan sulfate chains remains unchanged. Interestingly, TGF-β_1_ promotes the synthesis of perlecan in cultured bovine aortic endothelial cells without altering the length of the heparan sulfate chains if the cell density is high. The synthesis of biglycan with longer dermatan sulfate chains is also induced by TGF-β_1_. In contrast, IL-1α induces perlecan synthesis in other cell types, such as rabbit corneal stromal cells [30] and mouse hippocampal cells [31], although whether the length and disaccharide composition of heparan sulfate chains are altered by the cytokine is unknown. Recently, we found that 2,9-dimethyl-1,10-phenanthroline and dichloro(2,9-dimethyl-1,10-phenanthroline)zinc(II) modulate endothelial perlecan synthesis [25]. These two compounds increase perlecan synthesis in cultured bovine aortic endothelial cells when the cell density is high; however, the level of perlecan mRNA is unaffected by the compounds. The results from these series of studies indicate the absence of low molecular weight compounds that can selectively induce perlecan core protein synthesis without affecting heparan sulfate chain formation in vascular endothelial cells. In the present study, we found PMTAS as such a compound and propose that PMTAS may be a useful molecular probe to analyze the molecular mechanisms underlying the induction of endothelial perlecan core protein synthesis. Future works are needed to elucidate the intracellular signaling pathways that mediate the induction of endothelial perlecan synthesis by PMTAS and to confirm the activity of PMTAS in vivo. Additionally, proteoglycans derived from bovine and human endothelial cells have shown similar characteristics [32], and syndecan-4 which was found in rat endothelial cells, has also been expressed in bovine endothelial cells [33]. Therefore, the results of this study do not appear to be species dependent, however, this should be verified.

The variety and number of organic compounds in such hybrid molecules can be infinitely broadened by designing and synthesizing organic frames surrounding the central atom. However, very little is known about the biological activity of organic compounds containing high periodic main group elements. Consequently, we found an organoantimony compound, tris(pentafluorophenyl)stibine, as a transcriptional inducer of metallothionein (MT) in vascular endothelial cells, and analyzed the intracellular signal pathways that mediate the induction of MT isoforms [34]. In this study, we observed that tris(pentafluorophenyl)stibine increased the transcription of MT isoforms, MT-1A, and MT-2A to the same extent, whereas tris(pentafluorophenyl)phosphane only weakly caused transcriptional induction of MT-1A; however, it strongly induced that of MT-2A. This contrast is suggested to be due to differences in the location of the metal-responsive element region on the promoter of the MT isoform gene; to which the transcription factor metal-regulatory transcription factor (MTF)-1 is recruited [35]. These results indicate the importance of the intracellular metal(loid) atom in the structure of organic–inorganic hybrid molecules for their biological activities. Studies have been conducted on the cytotoxicity as well as the important role of the central atom in organic–inorganic hybrid molecules [3,4,7]. In the present study, we suggested that triphenylantimony structure induces the synthesis of perlecan, and the bridge containing the nitrogen atom in the PMTAS molecule intensifies the induction in vascular endothelial cells. The mechanisms of the structure–activity relationship for organic–inorganic hybrid molecules to exhibit biological activities are unclear; however, the three-dimensional structure of organic–inorganic hybrid molecules may be important for their biological activities, and their respective central atoms and substructures may contribute to their three-dimensional structures [36].

The present study revealed that PMTAS is an organic–inorganic hybrid molecule that induces the perlecan core protein synthesis in vascular endothelial cells independent of the cell density. We believe that this biological activity of PMTAS may be useful to analyze the molecular mechanisms underlying endothelial perlecan synthesis. Indeed, we have found the intracellular signal transduction pathways that mediate the expression of functional molecules, including MT, reactive sulfur species-producing enzymes, and proteoglycans. We revealed that a MT isoform MT-1A was induced by activation of both the MTF-1-metal responsive element (MRE) and nuclear factor-erythroid 2-related factor (Nrf2)- electrophile responsive element (ARE) pathways, whereas MT-2A expression only required the MTF-1-MRE pathway activation, using copper(II) bis(diethyldithiocarbamate) [37] and tris(pentafluorophenyl)stibine [34] as molecular probes. Further, we found that the extracellular signal-regulated kinase (ERK)1/2, p38 MAPK, and HIF-1α/HIF-1β pathways could mediate the transcription of cystathionine γ-lyase, a reactive sulfur species-producing enzyme, applying copper(II) bis(diethyldithiocarbamate) as a molecular probe [5]. We have previously shown that the HIF-1α/HIF-1β [23] and p38 MAPK pathways [24] mediate the expression of syndecan-4, a transmembrane heparan-sulfate proteoglycan, in vascular endothelial cells by employing 1,10-phenanthroline with or without zinc and copper(II) bis(diethyldithiocarbamate), respectively. Moreover, we believe that PMTAS is a suitable molecular probe to analyze the intracellular signal transduction pathways that mediate endothelial perlecan core protein synthesis. In addition, enhancement of PMTAS activity by chemical modification may be applied as a better tool to analyze the physiological and pathological roles of perlecan to study the regulation of vascular endothelial cell functions. Additionally, PMTAS may be a lead compound for drugs that exploit the biological activity of endothelial cell perlecan in drug discovery.

Although organometallic chemistry has developed rapidly, hybrid molecules have been mostly utilized as synthetic reagents, and their contribution to life sciences remains a future challenge. Importantly, there is a crucial difference between organic–inorganic hybrid molecules and organic/inorganic compounds. Specifically, the three-dimensional structure of organic structures can be drastically changed by the central atom [36]. In other words, the interaction between its central atom and organic structure can be utilized to target biological activities. In the PMTAS molecule, the antimony atom ensures a suitable three-dimensional structure to induce endothelial perlecan core protein synthesis. Thus, it also indicates that target molecules mediating endothelial perlecan core protein synthesis have a higher affinity to the whole structure of PMTAS. Additionally, it is demonstrated that the molecule is expressed in endothelial cells independent of cell density, whereas it is expressed in vascular smooth muscle cells only when the cell density is high. This is attributed to the inability of PMTAS to increase the accumulation of proteoglycans in vascular smooth muscle cells with a low cell density.

## 4. Materials and Methods

### 4.1. Materials

Bovine aortic endothelial cells and smooth muscle cells were purchased from Cell Applications (San Diego, CA, USA). Dulbecco’s modified Eagle’s medium (DMEM) and Ca^2+^- and Mg^2+^-free phosphate-buffered saline (CMF-PBS) were obtained from Nissui Pharmaceutical (Tokyo, Japan). Fetal bovine serum (FBS) was purchased from Biosera (Kansas City, MO, USA). PMTAS, DBMA, and SbPh_3_ were synthesized as reported previously [7] and diluted in dimethyl sulfoxide for experimental use. [^35^S]Na_2_SO_4_, Tran^35^S-label metabolic labeling reagent, and [methyl-^3^H]thymidine were obtained from MP Biomedicals (Santa Ana, CA, USA). L-[^14^C(U)]-Leucine was purchased from Moravek Biochemicals (Brea, CA, USA). Sepharose CL-4B, Sepharose CL-6B, and PD-10 columns were procured from GE Healthcare (Buckinghamshire, UK). Protease-free chondroitin ABC lyase (EC 4.2.2.4, derived from *Proteus vulgaris*), heparinase II (derived from *Flavobacterium heparinum*), and heparinase III (EC 4.2.2.8; derived from *Flavobacterium heparinum*) were purchased from Seikagaku (Tokyo, Japan). SbCl_3_, SbCl_5_, diethylaminoethyl-Sephacel (DEAE-Sephacel), Kodak XAR-2 film, and Immobbillon-P membranes were purchased from Merck KGaA (Darmstadt, Germany). Anti-perlecan antibody (sc-25848) was purchased from Santa Cruz Biotechnology (Santa Cruz, CA, USA). Horseradish peroxidase-conjugated anti-rabbit IgG (#7074) was obtained from Cell Signaling Technology (Beverly, MA, USA). The Chloride Assay Kit and Immunostar basic kit were obtained from Fujifilm Wako Pure Chemical Industries (Osaka, Japan). QIAzol lysis reagent was purchased from QIAGEN (Valencia, CA, USA). Luna Universal qPCR Master Mix was obtained from New England Biolabs Inc. (Ipswich, MA, USA). A high-capacity cDNA reverse transcription kit was purchased from Thermo Fisher Scientific (Waltham, MA, USA). Other reagents of the highest grade available were obtained from Nacalai Tesque (Kyoto, Japan).

### 4.2. Cell Culture and Treatments

Bovine aortic endothelial and smooth muscle cells were cultured separately in a humidified atmosphere of 5% CO_2_ at 37 °C in DMEM supplemented with 10% FBS until confluent. The cells were then transferred into 35 or 100 mm dishes or 24-well plates and cultured until confluent (“dense culture”) or into dishes or plates at a density of 5 × 10^3^ cells/cm^2^ and cultured for 24 h (“sparse culture”) in DMEM supplemented with 10% FBS. Subsequently, the medium was discarded, and the cells were washed twice with serum-free DMEM followed by treatment with PMTAS, SbCl_3_, SbCl_5_, DBMA, SbPh_3_, NPh_3_, PPh_3_, AsPh_3_, and BiPh_3_ (1, 5, or 10 µM each) for 4, 8, 12, 18, 24, or 48 h.

### 4.3. Incorporation of [^35^S]sulfate into Proteoglycans

Vascular endothelial cells and smooth muscle cells cultured in 24-well plates were treated with PMTAS, SbCl_3_, SbCl_5_, DBMA, SbPh_3_, NPh_3_, PPh_3_, AsPh_3_, and BiPh_3_ (1, 5, or 10 µM each) for 8, 24, or 48 h in the presence of [^35^S]sulfate (1 MBq/mL). The conditioned medium was harvested following treatment, and solid urea was added to yield a final concentration of 8 M. The cell layer was washed twice with 0.25 mL of ice-cold CMF-PBS and harvested by treatment with an 8 M urea solution containing 0.1 M 6-aminohexanoic acid, 5 mM benzamidine, 10 mM N-ethylmaleimide, 2 mM EDTA, 0.1 M phenylmethanesulfonyl fluoride, 0.1 M NaCl, 50 mM Tris base, and 2% Triton X-100 (pH 7.5), at 4 °C for 15 min. The cell and medium extracts were used to determine the [^35^S]sulfate incorporated into proteoglycans using the cetylpyridinium chloride (CPC) precipitation method [38], as described previously [28]. Briefly, aliquots of the extracts were spotted on filter papers and washed 5 times for 1 h in 1% CPC with 0.05 M NaCl. The radioactivity of the precipitated proteoglycans on the dried filter paper was measured via liquid scintillation counting.

### 4.4. Characterization of Proteoglycans

Vascular endothelial cells cultured in 100 mm dishes were treated with PMTAS (10 µM) for 48 h in the presence of [^35^S]sulfate (2 MBq/mL). After treatment, the cell and medium extracts were prepared as described above and chromatographed on PD-10 columns equilibrated in 8 M urea buffer (pH 7.5) containing 2 mM EDTA, 1 M NaCl, 0.5% Triton X-100, and 50 mM Tris base to obtain high molecular mass (>3 kDa) macromolecules. To separate proteoglycans based on their negative charges, the macromolecules were applied to a DEAE-Sephacel column (5 mL of resin) in 8 M urea buffer (pH 7.5) containing 2 mM EDTA, 0.1 M NaCl, 0.5% Triton X-100, and 50 mM Tris base. Unbound radioactive tags were removed from the column by washing with 30 mL of buffer. Bound radioactive molecules were eluted from the column with a linear gradient of 0.25–0.7 M NaCl in urea buffer (total volume of 50 mL). The radioactivity and concentration of NaCl in each fraction were measured using a liquid scintillation counting and chloride assay kit, respectively. The peaks eluted by approximately 0.4 M NaCl were pooled and concentrated by the application of the diluted samples to 0.3 mL DEAE-Sephacel minicolumns and elution of bound radioactivity with sequential washes of 8 M urea buffer containing 3 M NaCl. Subsequently, the samples were chromatographed on a Sepharose CL-4B column (0.9 × 80 cm) in 8 M urea buffer containing 0.25 M NaCl, and the radioactivity of each fraction was measured via liquid scintillation counting. The void and total volumes were estimated based on the elution positions of dextran blue and phenol red, respectively. Proteoglycan-containing peaks eluted at positions I and II were pooled and concentrated using DEAE-Sephacel minicolumns as described above. The proteoglycans in concentrated samples were precipitated with 3.5 volumes of 1.3% potassium acetate in 95% ethanol and 80 µg/mL carrier chondroitin sulfate for 2 h at −20 °C. The precipitation was repeated thrice. The precipitated proteoglycans were digested either with both heparinase II and heparinase III (0.03 U/mL each) in 0.2 M Tris–HCl buffer (pH 7.0) containing 10 mM calcium acetate or with 1.7 U/mL chondroitin ABC lyase in 50 mM Tris–HCl buffer (pH 8.0) containing 0.1 mg/mL BSA and 3 mM sodium acetate at 37 °C for 4 h or with 10 µg/mL papain in 0.1 M acetate buffer (pH 7.0) containing 5 mM EDTA and 5 mM cysteine at 65 °C for 4 h. The digested samples were chromatographed on a Sepharose CL-6B column (0.9 × 80 cm) in Tris-HCl buffer (pH 7.0) with 0.2 M NaCl, and the radioactivity of each fraction was measured by liquid scintillation counting. The void and total volumes were estimated based on the elution positions of dextran blue and phenol red, respectively.

### 4.5. DNA and Protein Synthesis

Vascular endothelial cells in 6-well culture plates were incubated in the presence or absence of PMTAS (10 µM) for 24 h and labeled with [^3^H]thymidine (10 kBq/mL) and [^14^C]leucine (50 kBq/mL) during the last 6 h of incubation. After incubation, the medium was discarded and the cells were washed twice with CMF-PBS, followed by collection with a rubber policeman in the presence of 0.75 mL of CMF-PBS. The culture well was washed with 0.75 mL of CMF-PBS, and the resulting wash was combined with the cell suspension. The cell homogenate was prepared by sonication, and the incorporation of [^3^H]thymidine or [^14^C]leucine into the 5% trichloroacetic acid-insoluble fraction of an aliquot of the cell homogenate was determined by liquid scintillation counting. A portion of the cell homogenate was used to determine the DNA content by the fluorometric method [39].

### 4.6. Analysis of Disaccharide Composition of Heparan Sulfate Chains

Heparan sulfate chains were extracted from the cell layer and conditioned medium of vascular endothelial cells, and their disaccharides were separated as in our previous experiment [29]. Briefly, vascular endothelial cells were treated with PMTAS (10 µM) for 48 h followed by proteoglycan extraction from the cell layer and conditioned medium under dissociative conditions in the presence of 8 M urea. The extract was concentrated on 0.3 mL DEAE-Sephacel minicolumns and precipitated with 3.5 volumes of 1.3% potassium acetate in 95% ethanol. The dried precipitate was digested overnight with proteinase K (800 µg/mL) in 0.1 M sodium acetate buffer (pH 7.2) at 60 °C. The glycosaminoglycan chains recovered with Microcon YM3 (3000 MW cut-off) ultrafiltration devices (Millipore, Billerica, MA, USA) were digested at 37 °C for 8 h with a mixture of heparinase II and heparinase III (0.03 U/mL each). The dried heparan sulfate samples were fluorotagged with 2-aminoacridone hydrochloride (0.1 M). The fluorotagged heparan sulfate hydrolase products were separated via electrophoresis, and the fluorescent images were displayed on a gel documentation system AE-6914 by ATTO (Tokyo, Japan). The bands of heparan sulfate unsaturated disaccharides were quantitatively analyzed using the NIH Image Analysis Software (ImageJ version 1.53k) using the bands of unsaturated GlcA-GalNAc(6S) as standards.

### 4.7. Analysis of Perlecan Core Protein

Vascular endothelial cells grown in 100 mm dishes were treated with PMTAS (10 µM) for 48 h in the presence of Tran^35^S-label metabolic labeling reagent (2 MBq/mL). After treatment, the cell and medium extracts were chromatographed using PD-10 and DEAE-Sephacel minicolumns as described above. The concentrated samples were precipitated with 3.3 volumes of 1.3% potassium acetate in 95% ethanol. Subsequently, the precipitated proteoglycans were digested with a mixture of heparinase II and heparinase III or with chondroitin ABC lyase, and sodium dodecyl sulfate-polyacrylamide gel electrophoresis (SDS-PAGE) was performed at 20 mA constant current in 3% stacking gels and 50 mA constant current in acrylamide 4–12% gradient slab gels. The radiolabeled proteoglycan core proteins were visualized via autoradiography of the dried gel exposed to a Kodak XAR-2 film at −80°C. For Western blot analysis, the proteoglycans separated by SDS-polyacrylamide gel were transferred to Immobillon-P membrane at 2 mA/cm^2^ for 1 h. The membrane was blocked with 5% skim milk in 20 mM Tris-HCl buffer solution (pH 7.5) containing 150 mM and 0.1% Tween 20 followed by incubation for 1 h with a primary antibody against perlecan (diluted 1:200) at 25 °C. The membrane was washed and thereafter incubated with horseradish peroxidase-linked secondary antibody for 1 h at 25 °C. Immunoreactive bands were visualized using the Immunostar basic kit and scanned with a LAS 3000 Imager (Fujifilm, Tokyo, Japan).

### 4.8. Real-Time Reverse Transcription Polymerase Chain Reaction (RT-PCR)

Total RNA from vascular endothelial cells treated with PMTAS (10 µM) for 4, 8, 12, 18, or 24 h was extracted as described previously [40]. Complementary DNA was synthesized from the mRNA using a high-capacity cDNA reverse extraction transcription kit. Subsequently, RT-PCR was performed at a reaction volume of 10 µL sample per well using Luna Universal qPCR Master Mix with 1 ng cDNA and 0.1 µM primers in a CFX connect real-time PCR system (BioRad). Levels of perlecan and glyceraldehydes-3-phosphate dehydrogenase (GAPDH) mRNAs were quantified using the relative standard curve method. The sequences of the bovine gene-specific forward and reverse primers were as follows: perlecan, 5′-ATGGCAGCGATGAAGCGGAC-3′ (forward) and 5′-TTGTGGACACGCAGCGGAAC-3′ (reverse) [25]; GAPDH, 5′-AACACCCTCAAGATTGTCAGCAA-3′ (forward) and 5′-ACAGTCTTCTGGGTGGCAGTGA-3′ (reverse) [27]. The fold change in the intensity value of perlecan was normalized to that of GAPDH.

### 4.9. Statistical Analysis

Data were presented as mean ± S.E. of three or four samples and analyzed for statistical significance using Student’s *t*-test, Dunnett’s test, or Tukey’s method, as appropriate. *p* = values less than 0.01 were considered to be statistically significant.

## 5. Conclusions

In conclusion, we found that PMTAS is a selective inducer of perlecan core protein synthesis in vascular endothelial cells. Thus, PMTAS would be a useful tool for future studies on mechanisms underlying perlecan core protein synthesis in vascular cells, which is importantly involved in the progression of vascular lesions, such as those in atherosclerosis. Further studies using PMTAS will be needed to elucidate the mechanisms, in other words, the intracellular signal pathways that mediate endothelial perlecan synthesis. This study supports our hypothesis that organic–inorganic hybrid molecules are useful as molecular probes to analyze biological systems.

## Data Availability

Not applicable.

## Supplementary Materials

The following supporting information can be downloaded at: https://www.mdpi.com/article/10.3390/ijms24043656/s1.
