# Supplementary material for: Sb-Phenyl-N-methyl-5,6,7,12-tetrahydrodibenz[c,f][1,5]azastibocine Induces Perlecan Core Protein Synthesis in Cultured Vascular Endothelial Cells
Source: Int J Mol Sci. 2023 Feb 11;24(4):3656. doi: 10.3390/ijms24043656 (PMC9959368; doi:10.3390/ijms24043656)
Supplement: Supplementary file 1 [file ijms-24-03656-s001.zip › Table S1.pdf]

Table S1. Organoantimony compounds used in this study.

No.		Molecular Form/Name
PMTAS	$C_{21}H_{20}NSb$	*Sb*-Phenyl-*N*-methyl-5,6,7,12-tetrahydrodibenz[*c,f*][1,5]azastibocine
SbPh3	$C_{18}H_{15}Sb$	Triphenylstibane
DBMA	$C_{15}H_{17}N$	*N*-Methyldibenzylamine
1	$C_{14}H_{12}BrOSb$	*Sb*-Bromo-7,12-dihydrodibenzo[*c,f*][1,5]oxastibocine
2	$C_{14}H_{12}BrSSb$	*Sb*-Bromo-7,12-dihydrodibenzo[*c,f*][1,5]thiastibocine
3	$C_{14}H_{12}ClSSb$	*Sb*-Chloro-7,12-dihydrodibenzo[*c,f*][1,5]thiastibocine
4	$C_{15}H_{15}BrNSb$	*Sb*-Bromo-*N*-methyl-5,6,7,12-tetrahydrodibenz[*c,f*][1,5]azastibocine
5	$C_{15}H_{15}ClNSb$	*Sb*-Chloro-*N*-methyl-5,6,7,12-tetrahydrodibenz[*c,f*][1,5]azastibocine
6	$C_{15}H_{15}FNSb$	*Sb*-Fluoro-*N*-methyl-5,6,7,12-tetrahydrodibenz[*c,f*][1,5]azastibocine
7	$C_{16}H_{17}BrNSb$	*Sb*-Bromo-*N*-ethyl-5,6,7,12-tetrahydrodibenz[*c,f*][1,5]azastibocine
8	$C_{16}H_{18}NSb$	*Sb,N*-Dimethyl-5,6,7,12-tetrahydrodibenz[*c,f*][1,5]azastibocine
9	$C_{17}H_{19}ClNSb$	*Sb*-Chloro-*N*-isopropyl-5,6,7,12-tetrahydrodibenz[*c,f*][1,5]azastibocine
10	$C_{18}H_{21}BrNSb$	*Sb*-Bromo-*N*-*tert*-butyl-5,6,7,12-tetrahydrodibenz[*c,f*][1,5]azastibocine
11	$C_{18}H_{21}BrNSb$	*Sb*-Bromo-*N*-isobutyl-5,6,7,12-tetrahydrodibenz[*c,f*][1,5]azastibocine
12	$C_{19}H_{24}NSb$	*Sb*-Methyl-*N*-*tert*-butyl-5,6,7,12-tetrahydrodibenz[*c,f*][1,5]azastibocine
13	$C_{19}H_{26}NSbSi$	*Sb*-Trimethylsilylmethyl-*N*-methyl-5,6,7,12-tetrahydrodibenz[*c,f*][1,5]azastibocine
14	$C_{20}H_{17}BrNSb$	*Sb*-Bromo-*N*-phenyl-5,6,7,12-tetrahydrodibenz[*c,f*][1,5]azastibocine
15	$C_{20}H_{17}OSb$	*Sb*-Phenyl-7,12-dihydrodibenzo[*c,f*][1,5]oxastibocine
16	$C_{19}H_{23}NSb$	*Sb*-Bromo-*N*-cyclohexyl-5,6,7,12-tetrahydrodibenz[*c,f*][1,5]azastibocine
17	$C_{21}H_{20}NSb$	*Sb*-Phenyl-*N*-methyl-5,6,7,12-tetrahydrodibenz[*c,f*][1,5]azastibocine (PMTAS)
18	$C_{23}H_{20}NSb$	*Sb*-phenylethynyl-*N*-methyl-5,6,7,12-tetrahydrodibenz[*c,f*][1,5]azastibocine
19	$C_{24}H_{22}NSb$	*Sb*-phenylethynyl-*N*-ethyl-5,6,7,12-tetrahydrodibenz[*c,f*][1,5]azastibocine
20	$C_{24}H_{26}NSb$	*Sb*-Phenyl-*N*-*tert*-butyl-5,6,7,12-tetrahydrodibenz[*c,f*][1,5]azastibocine
21	$C_{25}H_{24}NSb$	*Sb*-phenylethynyl-*N*-isopropyl-5,6,7,12-tetrahydrodibenz[*c,f*][1,5]azastibocine
22	$C_{26}H_{26}NSb$	*Sb*-phenylethynyl-*N*-isobutyl-5,6,7,12-tetrahydrodibenz[*c,f*][1,5]azastibocine
23	$C_{28}H_{22}NSb$	*Sb*-phenylethynyl-*N*-phenyl-5,6,7,12-tetrahydrodibenz[*c,f*][1,5]azastibocine
24	$C_{28}H_{28}NSb$	*Sb*-phenylethynyl-*N*-cyclohexyl-5,6,7,12-tetrahydrodibenz[*c,f*][1,5]azastibocine
