# Supplementary figures and images for: Sb-Phenyl-N-methyl-5,6,7,12-tetrahydrodibenz[c,f][1,5]azastibocine Induces Perlecan Core Protein Synthesis in Cultured Vascular Endothelial Cells
Source: Int J Mol Sci. 2023 Feb 11;24(4):3656. doi: 10.3390/ijms24043656 (PMC9959368; doi:10.3390/ijms24043656)

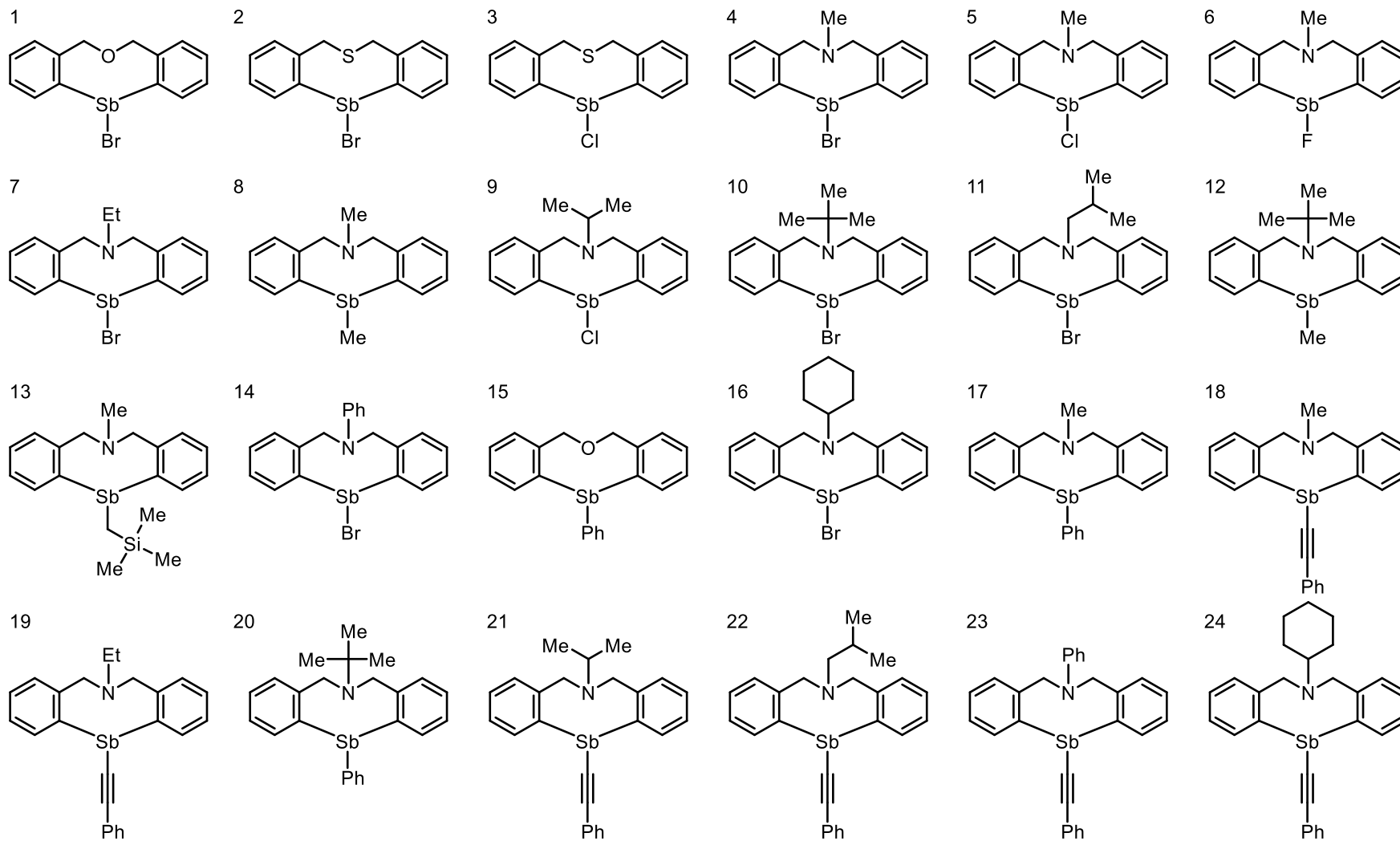

Supplement: Supplementary file 1 [file ijms-24-03656-s001.zip › Figure S1.pdf]
